# A Conservative Bioadhesive Approach to the Reattachment of Complicated Crown Fractures in Permanent First Molars: A Case Report with a 2-Year Followup

**DOI:** 10.1155/2012/256315

**Published:** 2012-02-09

**Authors:** Pragati Mirikar

**Affiliations:** Department of Conservative Dentistry and Endodontics, Sinhgad Dental College and Hospital, Maharashtra, Pune 411041, India

## Abstract

This paper presents a clinical report demonstrating combined restorative bioadhesive treatment and prosthetic rehabilitation of uncommon type of dental injury in an eighteen-year-old female involving crown fracture of all the permanent first molars and left upper premolars due to a bicycle riding accident. To restore the coronal fracture with invasion of biologic width, flap surgery with osteotomy and osteoplasty localized on the fractured teeth was performed, and the tooth remnant was reattached to the crown with a self-etch adhesive system. Frank pulp exposure was treated by self-etch dentin adhesive after surface disinfection prior to sealing of the wound site. At 2-year recall, the teeth continue to be aesthetically and functionally stable with a favourable pulpal and periapical environment.

## 1. Introduction

Fracture of crown with pulp exposure in a permanent tooth is a relatively uncommon injury. Andreasen reported that such fractures constitute about 5% to 8% of all traumatic injuries. The teeth may absorb the force of impact and fracture [1]. Tooth fragment reattachment techniques represent an important step in the science and art of restoring fractured anterior teeth. Despite the recent developments in adhesive materials and restorative techniques, there is no restorative material or technique that can reproduce the aesthetics and functional needs as well as the natural dental structure. Based on this, in clinical situations where the dental fragment is available and adequate for use, tooth fragment reattachment should be considered. It is a fine way to reinstate the natural shape, contour, surface texture, occlusal alignment, and colour of the fragment along with a positive emotional and social response from the patient to the preservation of natural tooth structure [2–7].

Restorative clinicians must understand the role of biologic width in preserving healthy gingival tissues and controlling the gingival form around restorations. When the fracture invades the biologic width and patient manages to save the tooth fragment, osteotomy and osteoplasty becomes necessary to determine the extent of fracture, to restore the biologic width, to gain access to the margins of tooth remnant, and to allow adequate isolation of the surgical field [3]. Literature is replete with spectrum of strategies utilized for reattachment of fractured dental fragments for restoration of anterior teeth [4, 5]; however, this paper is unique as paper it reports the occurrence and describes a systematic approach for evaluation and management of a patient with multiple posterior teeth fracture involving dislocation of several cusps and the use of dentin bonding adhesive for reliable reattachment of fractured teeth fragments.

## 2. Case Report

**Figure 1:**
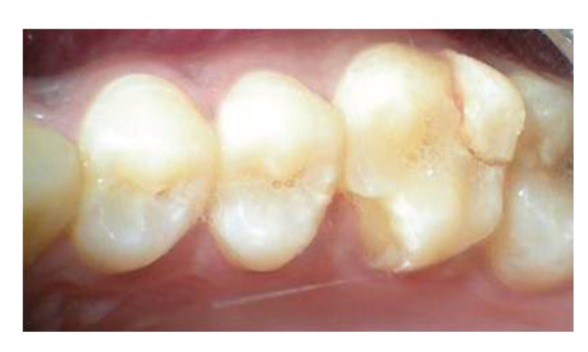
Oblique fracture of distofacial cusp with 16 with loss of mesiopalatal cusp with 16.

**Figure 2:**
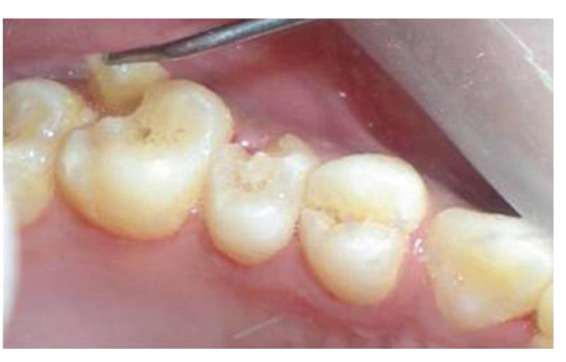
Oblique fracture of distofacial cusp with 26, loss of buccal cusp with 25.

**Figure 3:**
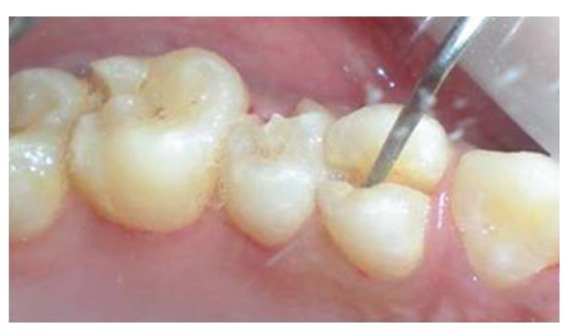
Mesiodistal fracture in the vertical direction with 24 extending subcrestally.

**Figure 4:**
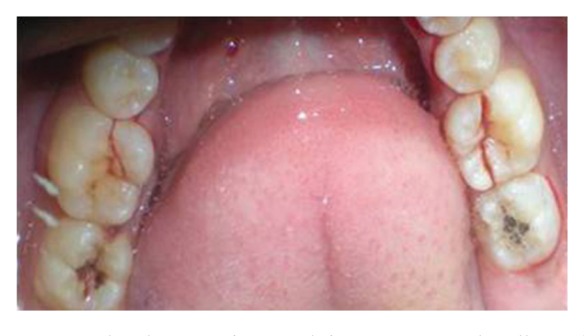
Occlusal view of vertical fracture mesiodistally with 36 and oblique fracture of the mesiolingual cusp with 46.

**Figure 5:**
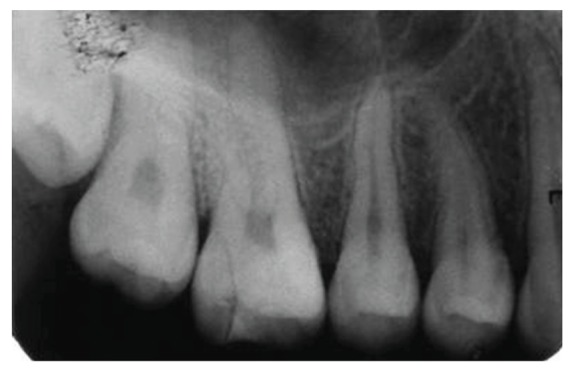
Preoperative radiograph revealing oblique fracture involving distal aspect of the tooth 16.

**Figure 6:**
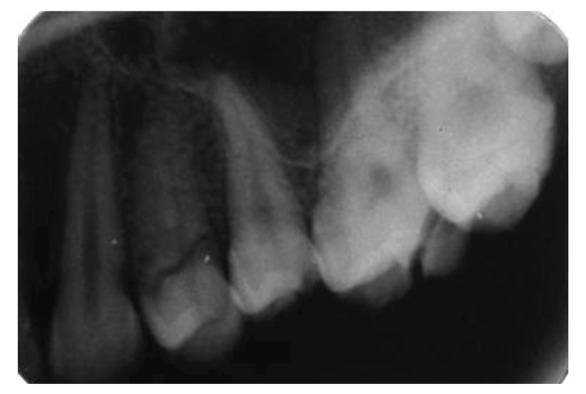
Preoperative radiograph revealing oblique root fracture with 24, loss of buccal cusp with 25, and oblique fracture of distobuccal cusp with 26.

**Figure 7:**
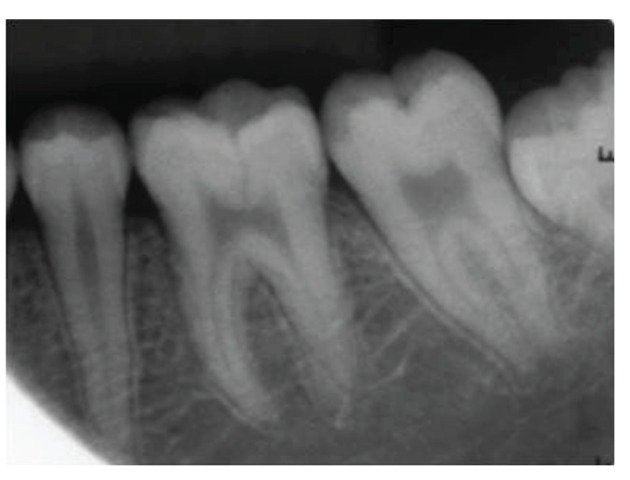
Preoperative periapical radiograph with 36 showing the radiolucent fracture line in the coronal area and passing through the pulp chamber.

**Figure 8:**
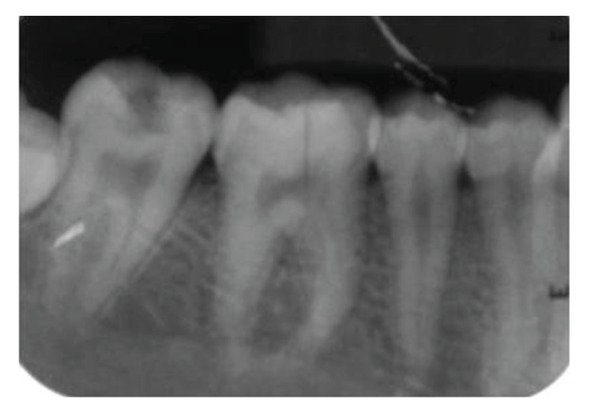
Preoperative periapical radiograph with 46 revealing radiolucent fracture line in the coronal aspect of the tooth.

The patient reported to the dental clinic with a chief complaint of fractured teeth in upper and lower back region of the jaw due to a bicycle riding accident. The general condition of the patient was assessed and recorded prior to a detailed examination of head, neck, and oral regions for damage of hard and soft tissues. After a thorough evaluation of the periodontal, endodontic, coronal, and occlusal (PECO) status of the teeth [6, 7], a diagnosis of oblique fractures with 16 (Figure 1), 26, 25 (Figure 2), and vertical fracture with 24 extending subcrestally (Figure 3) was made. With respect to the mandibular molars, oblique fracture with 46 involving mesiolingual cusp (Figure 4) and vertical fracture with 36 extending mesiodistally (Figure 4) were diagnosed. In addition to clinical examination for pulpal exposure, the vitality status of all the teeth was recorded as the baseline responses. The clinical diagnosis was confirmed radiographically (Figures 5, 6, 7, and 8), which also suggested the presence of transverse fracture at the level of coronal and middle third of the root with respect to 24 (Figure 6). Patient was informed of the difficulty of the case and was offered with several treatment alternatives and told about the need for exploratory surgery to define the best treatment modality [6, 7]. Consent was secured from the patient who was previously informed about eventual risks such practices involved. The aim was to preserve the greatest amount of supporting bone and to render rational treatment. Surgery was required to access fracture extension and to assert the viability of fragment reattachment. As a first step, antisepsis and anaesthesia of the involved teeth were carried out. Next fragments were tested for adaptation.

**Figure 9:**
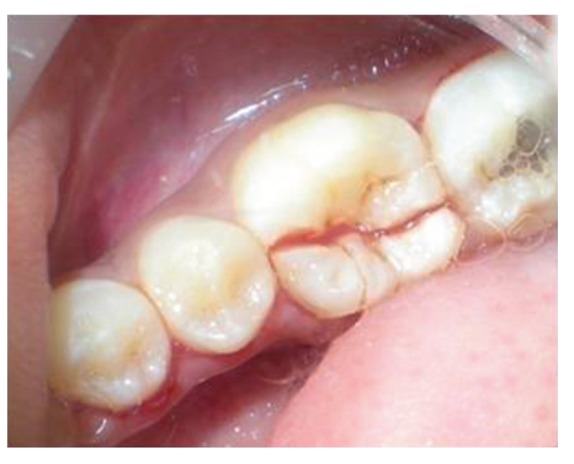
Preoperative view of 36, prior to placing surgical incisions for a full thickness mucoperiosteal flap on lingual aspect.

**Figure 10:**
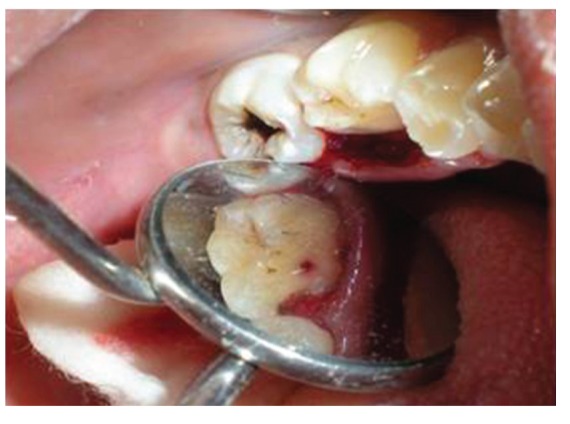
Lingual view of 36, revealing frank pulp exposure after removal of the partially attached dental fragments and surface disinfection with 0.12% chlorhexidine. Surgical site reveals the relationship of fracture to the osseous crest.

**Figure 11:**
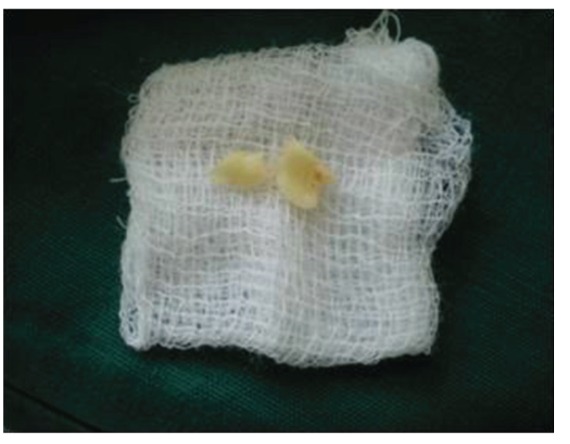
Buccal view of dental fragments of fractured 36, after surface disinfection with 0.12% chlorhexidine and application of dentin bonding adhesive.

**Figure 12:**
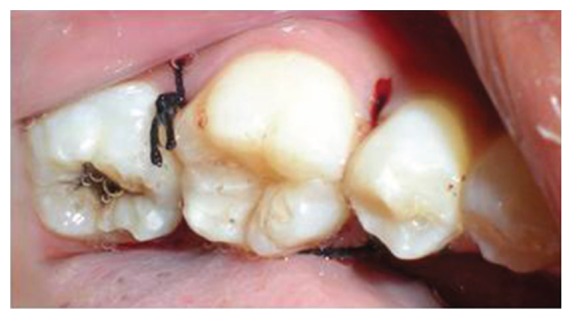
Completed immediate repair with 36 and sutures in place.

Surgical treatment was initiated with 36 (Figure 9). To gain access to the cervical margin of the dental remnant and thus better evaluate the relation to the bone crest, a full thickness flap was planned. An exploratory flap was made with a no. 15 scalpel blade, using lingual intrasulcular and vertical releasing incisions. The fracture had occurred in the mesiodistal dimension, dividing the lingual aspect of the tooth into mesial and distal halves and a pin point pulp exposure (Figure 10). The two fractured fragments that were partially attached with the help of gingival fibres were separated and were maintained in normal saline (Figure 11) [7]. The fracture line had invaded the biologic width and the need of osteotomy of about 1 mm on the lingual aspect was evident, so as to restore the dimensions of the biologic space [8]. After disinfection of both the tooth and the fractured fragment and with 0.12% CHX solution [7, 8], an adhesive system (Clearfill SE) was applied to dentin and enamel and to hybridize the conditioned surfaces on both the tooth remnant and the fragment [9, 10] in accordance with manufacturer's instructions. The operative procedure was performed in a moisture-free field, which was maintained with the help of high volume suction and cotton roll isolation [3]. Since there were two fragments present, it was necessary to assemble the pieces with resin composite prior to trial in mouth [11]. Excess adhesive was removed with mild air jet. Usually, at this stage, polymerization would be the next step; however, in an effort to attain an adequate repositioning of fragment on remnant, light polymerization was not conducted, because the light-cured adhesive would make it impossible to seat the fragment correctly [11]. A microfilled flowable composite resin (A3, Flowable, 3 M ESPE) was used to perform attachment [12]. After receiving a slight layer of resin, the fragment was repositioned and kept in position until light polymerization was completed. The surgical site was closed, and interrupted sutures were placed (Figure 12).

**Figure 13:**
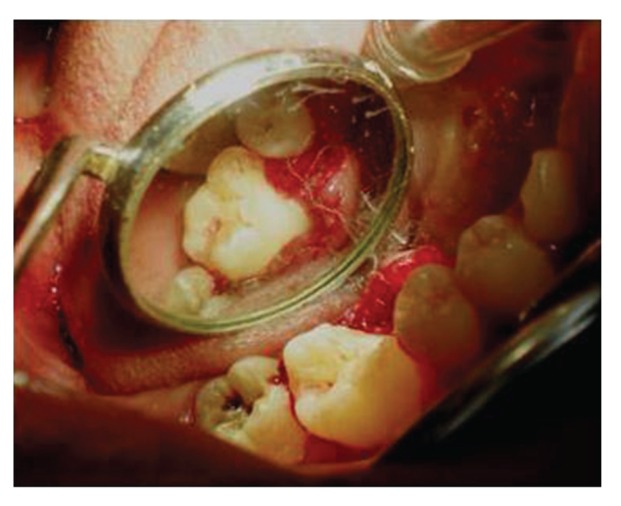
Lingual view of reflected full thickness mucoperiosteal flap and surgical correction of biologic width with 46.

**Figure 14:**
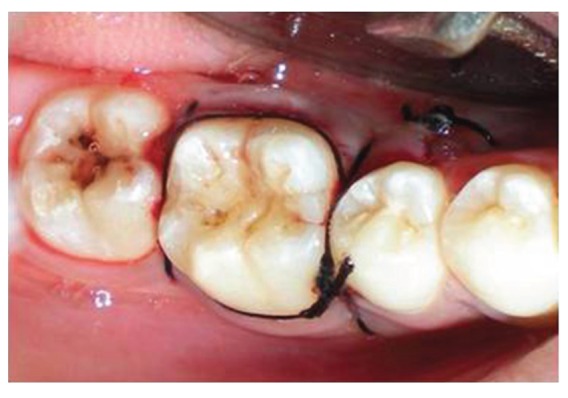
Clinical view after the fragment attachment has been performed, and flaps have been sutured.

The severity of fracture in the subgingival direction was the most important variable influencing treatment planning even with 46 [3, 6]. Although the invasion of biologic width was small in extent and magnitude, a similar surgical approach was executed so as to reattach the fractured mesiolingual fragment (Figures 13, and 14).

**Figure 15:**
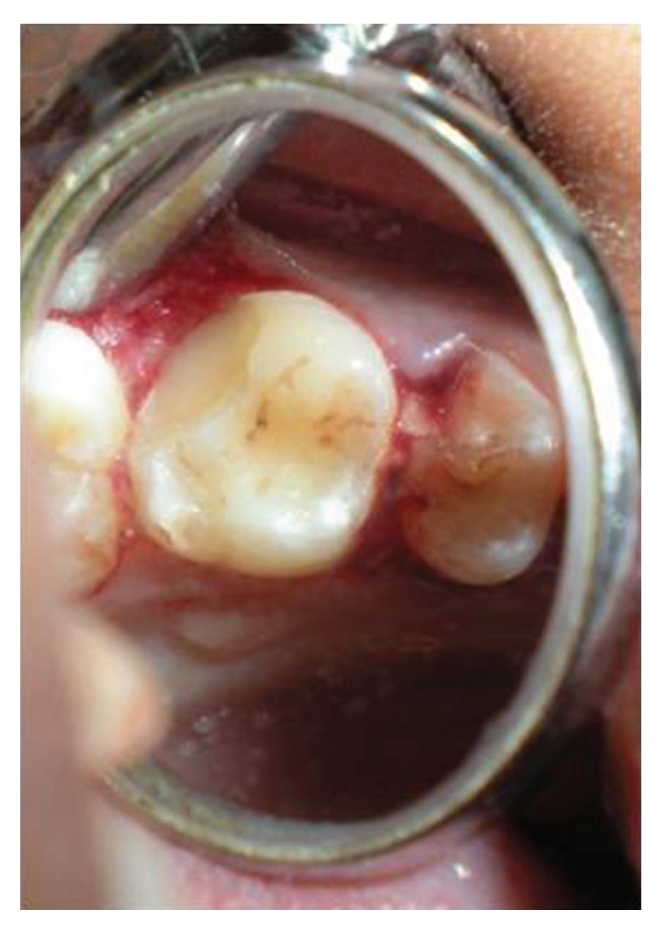
Buccal view of obliquely fractured 26 and 25, after reflection of the full thickness mucoperiosteal flap on the facial aspect. Relationship of fracture to remaining gingival tissues.

**Figure 16:**
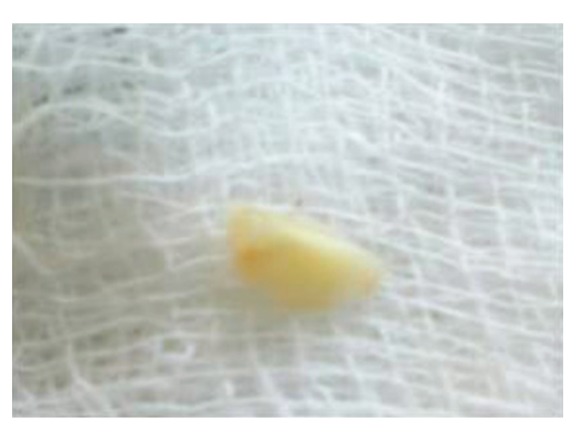
Fractured fragment of distofacial cusp of 26 after surface disinfection and application of bonding agent. Fragment demonstrates oblique nature of fracture.

**Figure 17:**
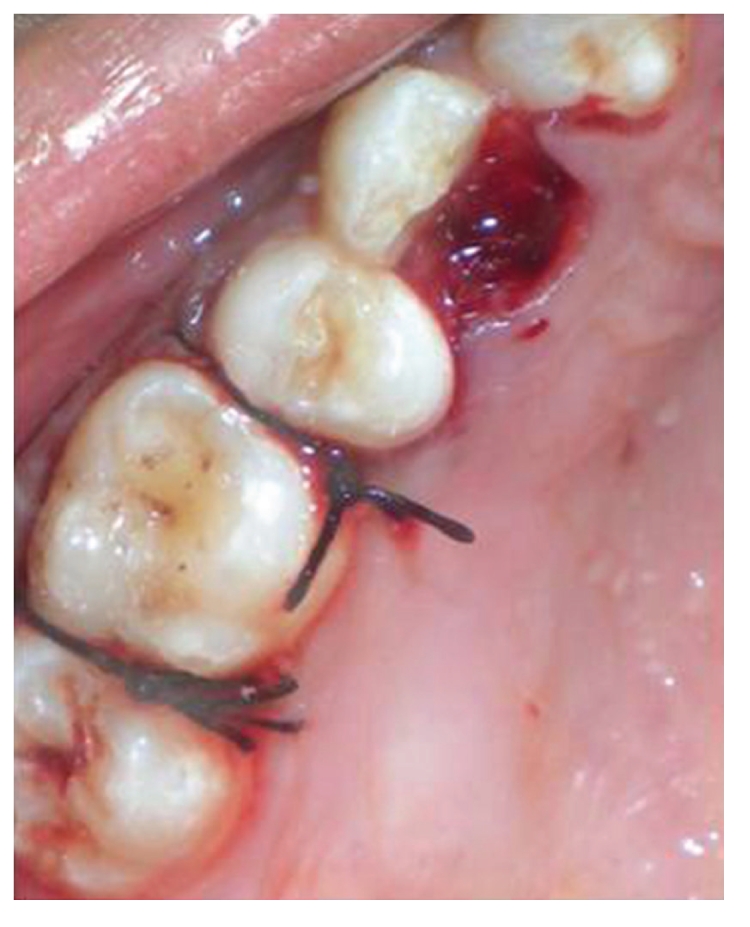
Restoration and reattachment of fractured fragments of 25 and 26. Occlusal view of 24 not amenable to restoration and hence subsequently extracted.

With respect to the left quadrant of the upper arch, a full thickness mucoperiosteal flap was reflected in the region of 24, 25, and 26 (Figure 15), and the procedure for reattachment with the obliquely fractured distofacial cusp of 26 was performed as is described previously (Figures 16 and 17). The restoration of the fractured and lost buccal cusp of 25 was done with microhybrid restorative resin (Figure 17). In this case, the healthy tooth margins were in enamel and were placed supragingivally, which provided a reliable mean of bonding. 24 was extracted, considering there were multiple fragments, which were practically impossible to juxtrapose and reattach (Figure 17). In addition, due to root fracture at the junction of coronal and middle third, a surgical attempt to restore the biologic width would have led to a substantial amount of loss of the supporting alveolar bone.

**Figure 18:**
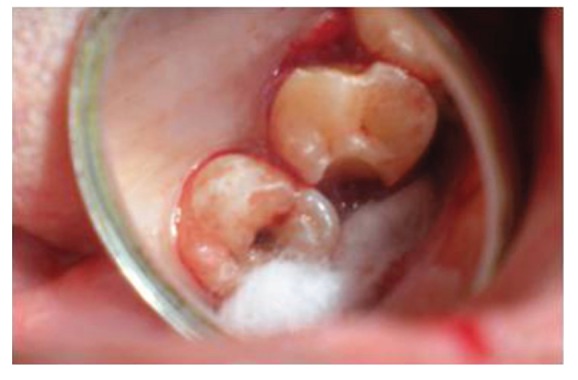
Occlusal view of surgical site of fractured 16 after surface disinfection and application of bonding agent.

**Figure 19:**
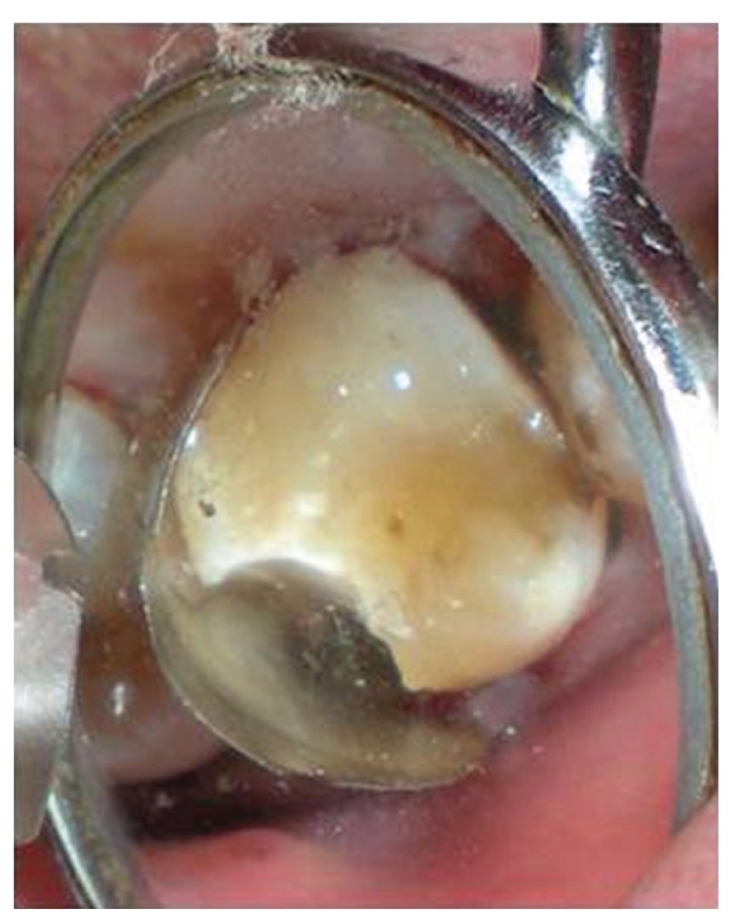
Occlusal view of 16 after application of dead soft matrix band for accurate positioning of distofacial cusp and restoration of the lost mesiopalatal cusp with restorative resin.

**Figure 20:**
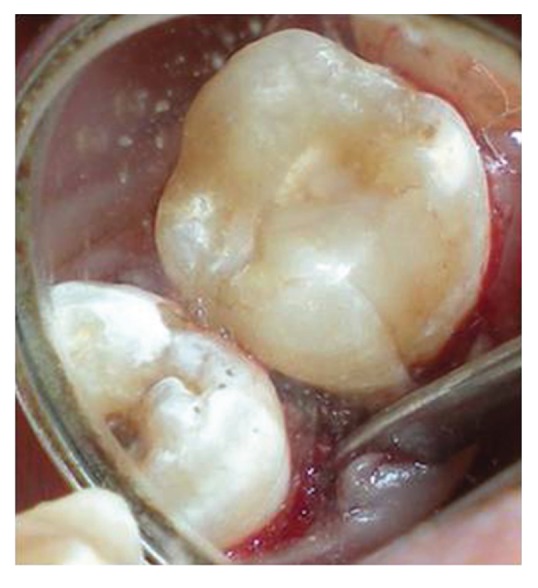
Occlusal view of fractured distofacial fragment positioned at its original site with respect to 16. Build up of lost mesiopalatal fragment is done with microhybrid resins.

**Figure 21:**
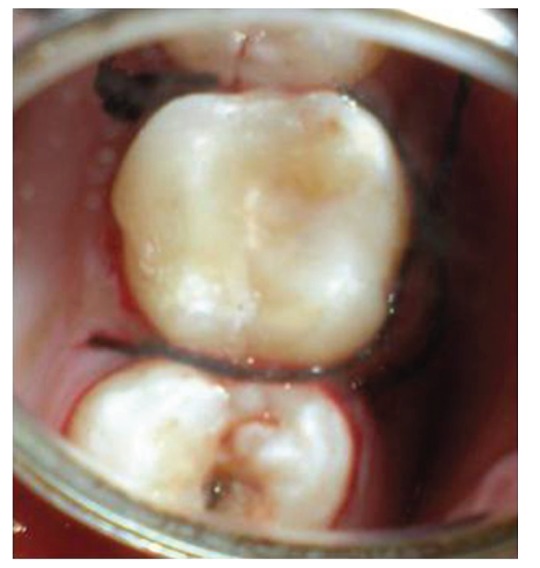
Closure of the surgical site with sutures in place.

 In case of 16, the procedure for reattachment for the fractured distofacial cusp was performed in a similar way (Figure 18). There was loss of mesiopalatal cusp, when tooth was fractured and hence it was decided to restore the lost dental fragment with composite resin. The first increment was of microhybrid type, to be covered with a microfilled resin to attain greater surface smoothness and finish (Figure 19) [11]. These increments were photopolymerized, and the restoration finished and polished (Figure 20). Surgical site was closed with sutures (Figure 21).

**Figure 22:**
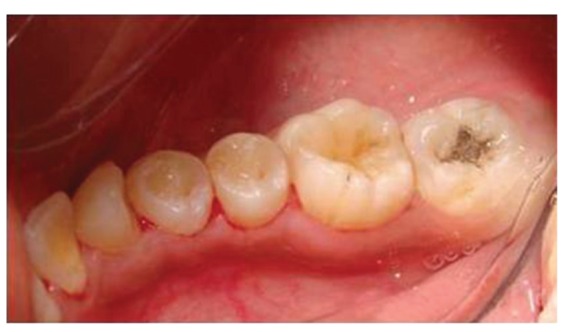
Lingual view of 46, seven days after reattachment and after suture removal.

**Figure 23:**
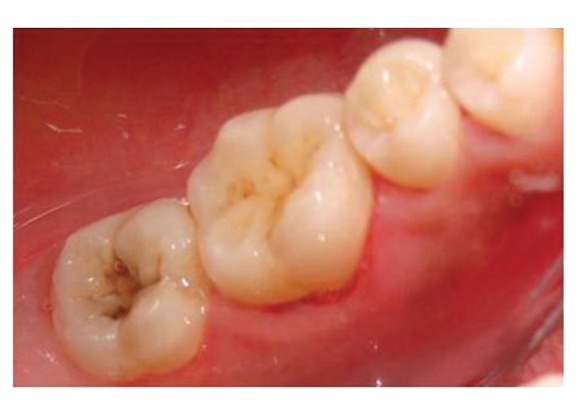
Lingual view of 36, seven days after reattachment with favourable periodontal response.

**Figure 24:**
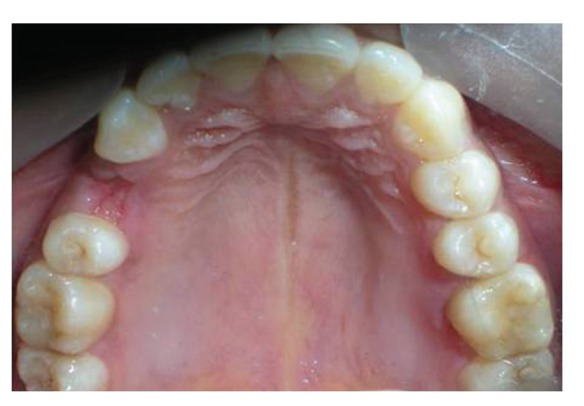
occlusal view of upper arch, seven days posttreatment showing excellent periodontal healing.

**Figure 25:**
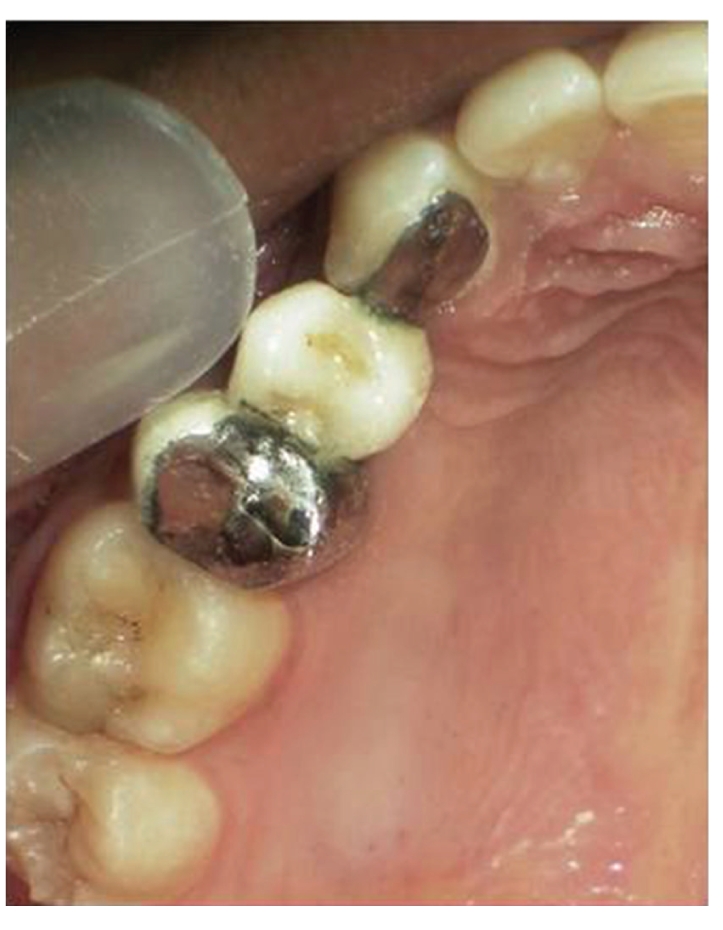
Luted in place porcelain fused to metal prosthesis fabricated by a conservative approach to replace 24.

Patient was recalled after seven days for suture removal. Immediate results at 7 days revealed a stable reattachment (Figures 22, 23, and 24). The tissues were undergoing healing. A conservative approach was planned for prosthetic rehabilitation of 24, with a full coverage PFM crown with porcelain facing for 25 and wings on palatal aspect of canine. Impression was made with polyvinyl siloxane impression material and self-cure acrylic resin temporaries were placed. Cementation was done with dual cure resin luting cement Calibra (Coltene Whaledent) (Figure 25) [13, 14].

**Figure 26:**
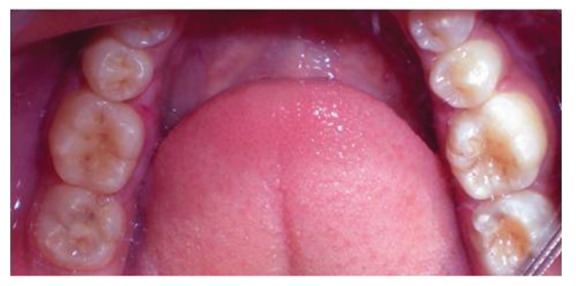
Occlusal view of the mandibular arch at 6-month recall shows satisfactory healing.

**Figure 27:**
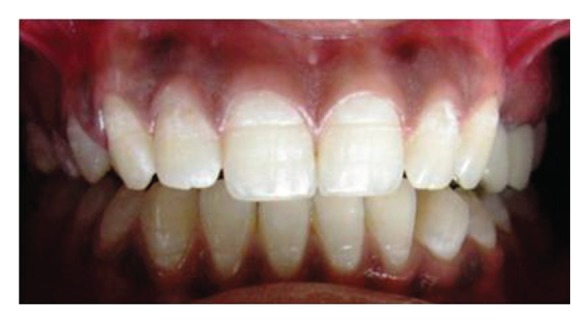
Frontal view at 6-month recall showing functional and aesthetic harmony.

The patient was recalled periodically after reattachment. Posttreatment photographs of treated teeth at an interval of six months demonstrate functional and aesthetic harmony with the adjacent oral tissues (Figures 26, and 27).

**Figure 28:**
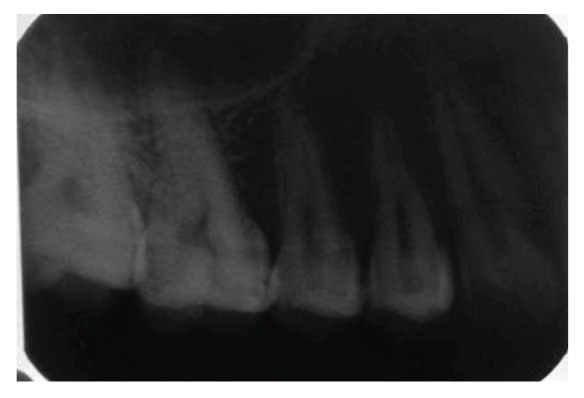
Intraoral periapical radiograph with 16 at 2-year followup.

**Figure 29:**
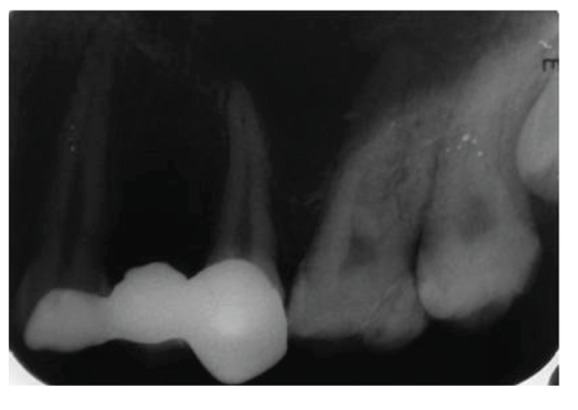
Intraoral periapical radiograph with upper left posterior region at a 2-year followup.

**Figure 30:**
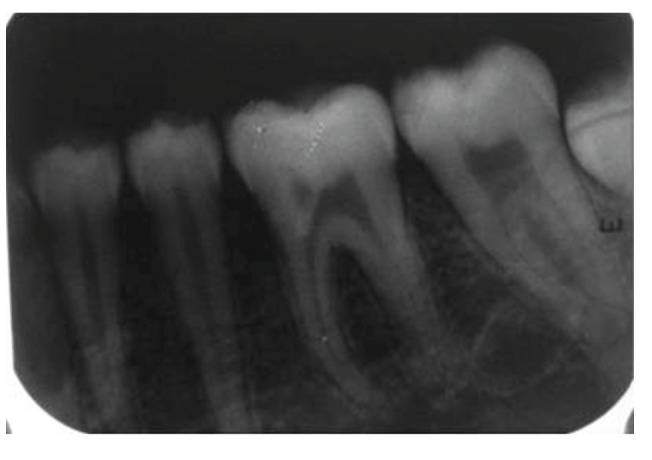
Intraoral periapical radiograph with 36 at 2-year followup. No significant pulpal or periapical changes.

**Figure 31:**
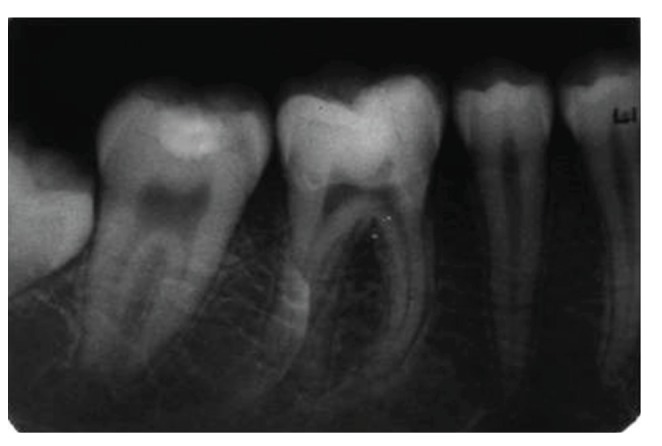
Intraoral periapical radiograph with 46 at 2 years followup. No significant pulpal or periapical changes.

**Figure 32:**
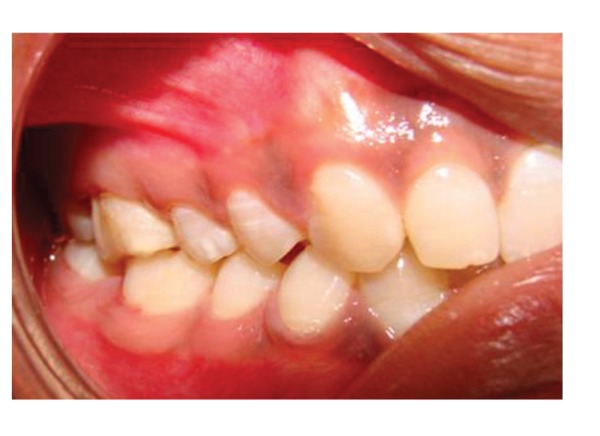
Right lateral view of teeth in functional harmony 2 years posttreatment.

**Figure 33:**
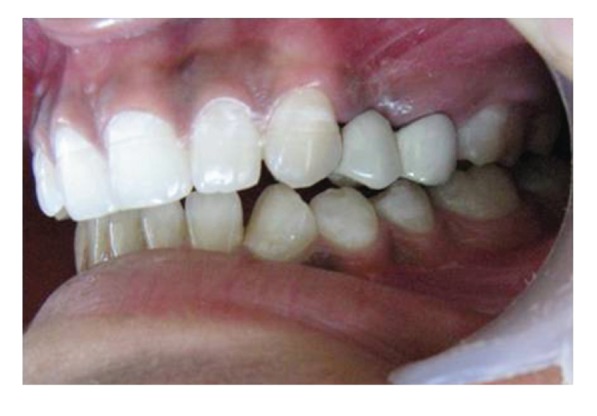
Left lateral view of teeth in functional harmony 2 years postoperative.

A follow-up examination of periodontal, pulpal, and occlusal status for a period of two years was done. Radiographic examination revealed no significant pulpal or periapical changes (Figures 28, 29, 30, and 31). No alterations that could jeopardize the treatment were observed at the periodontium. The teeth continue to be in functional and aesthetic harmony (Figures 32 and 33).

## 3. Discussion

Traumatic lesions range from simple lesions, involving only enamel, to more complex lesions in which pulpal and periodontal tissues are involved [1]. Esthetic, biologic, and restorative problems may occur as a result of fracture extending subgingivally and impinging on the biologic width [3, 11, 15]. The treatment option depends on the relationship of the fracture to the alveolar crest [14, 16], degree of pulpal involvement, [17, 18] extent of apex formation, and aesthetic requirements of the patient [11].

Treatment alternatives included crown lengthening, flap surgery and ostectomy/osteoplasty to restore biologic width, followed by crown reattachment and rapid orthodontic root extrusion possibly in conjunction with fibrotomy [6, 19].

A healthy coexistence between teeth and their surrounding periodontal structure is the goal of the conscientious dentist and the expectation of informed patient. Reattachment of tooth fragments is a viable alternative to conventional resin bonding or fixed prosthodontics. With the evolution of contemporary resin adhesive systems [9, 20–22], which allow strong durable bond to dentin, reattachment of dental fragments has been shown to be noninvasive treatment offering good results, even when performed under challenging conditions [8, 20].

The decision not to adopt any kind of chamfer on either side of the fragment or tooth remnant was one modified from that suggested by Dean et al. [23], in which they concluded that there was no difference in the fracture strengths of fragments that received no preparation. In spite of the large number of publications presenting different approaches to the preparation of tooth fragments, there are no reports of the long-term effects of such preparations. Bevels, chamfers [24–26], grooves intraenamel “V” shaped notches [2, 4], and undercuts have been proposed, but these modifications can adversely affect the accurate positioning of tooth fragment and apparently make no difference in the prognosis. Provided that minimal damage to the fragment has occurred, simple disinfection of the tooth and its fragment with 0.12% chlorhexidine solution is effective before apposition [7].

Since it has been proved that greatest threat to the pulpal vitality is bacteria and not dental materials, disinfection of the fracture site and the fragment was considered imperative [27, 28]. Storage in normal saline is recommended as this will minimize any dimensional change [7]. As important as the level of hydration of the fragment is its adequate adaptation to the dental remnant. The technique becomes complicated when multiple fragments exist, because they must first be bonded to each other, whenever possible, before reattachment [11, 12].

Whenever the fracture invades the biologic width and the invasion is of small extent and magnitude, flap surgery should be performed, with minimal osteotomy and osteoplasty and if at all possible, without involving adjacent teeth. This is followed by the reattachment of the tooth fragment [3]. Restoration techniques both simple and complex; must endeavour to preserve periodontal health. Longevity of restoration and tissue health maintenance are, after all, the best evidence of success for any restorative treatment [3, 10, 29].

The addition of resin becomes fundamental in cases in which a perfect fragment to tooth adaptation is not obtained or the resin-tooth line is too evident [10]. Studies have demonstrated a good prognosis when pin point exposures are treated with adhesive systems. Such studies are based on the fact that the seal offered by adhesives protects the pulp against penetration of bacteria, preventing pathologic alterations [30].

Longevity of a tooth fragment reattachment is not foreseeable, but the real merit of reattachment is the fact that all other restorative options, such as direct adhesive ones, veneers, and crowns will always be open. With advancement in dental bonding technology, it is now possible to achieve excellent results with reattachment of dislocated tooth fragments, provided that the biologic factors and selection of materials are logically assessed and managed.

## 4. Conclusion

When young patients with tooth fracture are treated, with a conservative treatment modality, the reattachment of teeth fragments through adhesive techniques, even when the fracture is severe, can be considered a safe procedure with predictable results, provided that cases are selected judiciously. Emphasis was being given to those techniques that restore biologic width. These clinical experiences also support the notion that bacteria, not materials, are the greatest threat to tooth vitality and the proper sealing of the tooth is of great importance to us so as to achieve favorable long-term prognosis.
